# Optogenetic Stimulation of the Basolateral Amygdala Increased Theta-Modulated Gamma Oscillations in the Hippocampus

**DOI:** 10.3389/fnbeh.2019.00087

**Published:** 2019-04-30

**Authors:** Nathan S. Ahlgrim, Joseph R. Manns

**Affiliations:** ^1^Graduate Program in Neuroscience, Emory University, Atlanta, GA, United States; ^2^Department of Psychology, Emory University, Atlanta, GA, United States

**Keywords:** hippocampus, amygdala, memory, optogenetics, oscillations, theta, gamma

## Abstract

The amygdala can modulate declarative memory. For example, previous research in rats and humans showed that brief electrical stimulation to the basolateral complex of the amygdala (BLA) prioritized specific objects to be consolidated into long term memory in the absence of emotional stimuli and without awareness of stimulation. The capacity of the BLA to influence memory depends on its substantial projections to many other brain regions, including the hippocampus. Nevertheless, how activation of the BLA influences ongoing neuronal activity in other regions is poorly understood. The current study used optogenetic stimulation of putative glutamatergic neurons in the BLA of freely exploring rats to determine whether brief activation of the BLA could increase in the hippocampus gamma oscillations for which the amplitude was modulated by the phase of theta oscillations, an oscillatory state previously reported to correlate with good memory. BLA neurons were stimulated in 1-s bouts with pulse frequencies that included the theta range (8 Hz), the gamma range (50 Hz), or a combination of both ranges (eight 50-Hz bursts). Local field potentials were recorded in the BLA and in the pyramidal layer of CA1 in the intermediate hippocampus. A key question was whether BLA stimulation at either theta or gamma frequencies could combine with ongoing hippocampal oscillations to result in theta-modulated gamma or whether BLA stimulation that included both theta and gamma frequencies would be necessary to increase theta–gamma comodulation in the hippocampus. All stimulation conditions elicited robust responses in BLA and CA1, but theta-modulated gamma oscillations increased in CA1 only when BLA stimulation included both theta and gamma frequencies. Longer bouts (5-s) of BLA stimulation resulted in hippocampal activity that evolved away from the initial oscillatory states and toward those characterized more by prominent low-frequency oscillations. The current results indicated that one mechanism by which the amygdala might influence declarative memory is by eliciting neuronal oscillatory states in the hippocampus that benefit memory.

## Introduction

The basolateral complex of the amygdala (BLA) is a key modulatory region of hippocampus-dependent memory (McGaugh, 2002). Direct activation of the BLA via pharmacological manipulations (Roozendaal et al., 2008; Barsegyan et al., 2014) or brief electrical stimulation (Bass et al., 2012, 2014; Bass and Manns, 2015; Inman et al., 2018) improved performance in memory tasks not designed to be overtly emotional, such as object recognition memory tasks. Indeed, in one recent study with human participants, direct electrical stimulation targeting the BLA improved recognition memory for neutral object images despite participants reporting that they could not detect the stimulation (Inman et al., 2018). These experiments built on prior work in rodents demonstrating that the BLA mediated the influence of emotional arousal on memory performance in tasks such as inhibitory avoidance (McIntyre et al., 2002, 2005; McReynolds et al., 2010, 2014b; Holloway-Erickson et al., 2012; Huff et al., 2013). Thus, existing research suggests that activation of the BLA can modulate memory for the better and can be engaged by emotional arousal or by direct intervention.

The BLA is thought to modulate memory in part by influencing memory processes in other brain regions (McGaugh, 2002; Roozendaal et al., 2003, 2006). In particular, the BLA sends direct glutamatergic projections to the hippocampus and to regions such as the entorhinal and perirhinal cortices that in turn project to the hippocampus (Pitkänen et al., 2000). Inactivating the hippocampus via local infusion of muscimol blocked the object recognition memory improvement triggered by electrical stimulation of the BLA (Bass et al., 2014), whereas pharmacological manipulations of the BLA such as local infusion of adrenergic agonists led to increased markers of synaptic plasticity in the hippocampus (McIntyre et al., 2005; McReynolds et al., 2014a). In addition, electrical stimulation of the BLA increased slow gamma oscillatory activity in the hippocampus (Bass and Manns, 2015). Many brain regions receive inputs from the BLA (Sah et al., 2003), but for modulation of hippocampus-dependent memory, the current data suggest one key region influenced by the BLA is the hippocampus itself. Understanding these mechanisms will help characterize how the brain prioritizes important memories (Manns and Bass, 2016).

One possible mechanism by which the BLA could beneficially modulate memory is by eliciting oscillatory network states that favor memory in the hippocampus and associated areas. In particular, theta (6–10 Hz in rats) oscillations are related to behavioral states (Montoya et al., 1989; Sheremet et al., 2019) and memory (Buzsáki, 2005; McNaughton et al., 2006; Buzsaki and Moser, 2013). In addition, hippocampal slow gamma oscillations (30–55 Hz in rats) at encoding correlated with later retrieval success (Sederberg et al., 2007; Jutras et al., 2009; Trimper et al., 2017). The amplitude of slow gamma oscillations in the hippocampus fluctuates and is modulated by the phase of theta, one type of phase-amplitude cross-frequency coupling (hereby referred to as theta–gamma comodulation). The degree of theta–gamma comodulation is also a strong correlate of memory performance (Tort et al., 2009; Shirvalkar et al., 2010; Trimper et al., 2014). Indeed, recent studies using electrical stimulation to the BLA to enhance object recognition memory have used an electrical pulse frequency meant to simulate theta-modulated gamma oscillations (bursts of 50 Hz stimulation every 1/8th of a second; Bass et al., 2012, 2014; Bass and Manns, 2015; Inman et al., 2018). These results indicated that stimulating the BLA with a theta-modulated gamma pulse frequency was capable of improving memory performance, but the findings did not answer whether stimulating at theta or gamma frequencies alone would suffice to elicit in the hippocampus neuronal oscillations resembling those that correlate with good memory. For example, stimulating the BLA at 50 Hz alone could in principle lead to slow gamma (i.e., 50 Hz) oscillations in the hippocampus for which the amplitude would be modulated by the phase of the endogenous hippocampal theta oscillations.

The current experiment with freely moving rats asked if stimulating the BLA at theta and gamma frequencies could elicit in the hippocampus neuronal oscillations resembling those previously found to correlate with good memory performance. A key question was whether BLA stimulation that combined theta and gamma frequencies was needed to amplify hippocampal theta–gamma comodulation, which is known to be important for good memory. The current experiment utilized optogenetic rather than electrical stimulation of the BLA for several reasons. First, the use of a cell-type specific (CaMKII) promoter for the vector delivering the opsin (channelrhodopsin; ChR2) allowed for stimulation restricted to putative glutamatergic projection neurons in the BLA. Second, use of a blue light-sensitive opsin allowed for a control stimulation condition that used near-infrared light pulses outside the excitation spectrum of the opsin. Third, optical stimulation avoided electrophysiological recording artifacts induced by electrical stimulation. Stimulation was delivered in 1-s bouts at 8 Hz to emulate theta, at 50 Hz to emulate slow gamma, and at 50 Hz bursts every 1/8th second to emulate theta-modulated gamma (50/8 Hz). Included for comparison were conditions in which 1 s of 20 Hz stimulation was delivered using either blue (experimental) and near-infrared (control) light. BLA stimulation with blue light in all conditions elicited oscillatory activity in the hippocampus, but only BLA stimulation at 50/8 Hz elicited in CA1 a pattern of activity that appeared to reflect theta–gamma comodulation similar to what has been observed in studies to positively correlate with good object memory (Tort et al., 2009; Shirvalkar et al., 2010; Trimper et al., 2014).

## Materials and Methods

### Subjects

Six adult male Long-Evans rats, between 400 and 500 g, were housed individually (12-h light/dark cycle; stimulation during light phase). All animals were given free access to water and were food restricted, maintaining at least 90% of their free-feeding body weight. All procedures involving rats were approved by the Institutional Animal Care and Use Committee at Emory University.

### Surgery and Drive Positioning

Rats underwent a single stereotaxic surgery for infusion of the viral vector and implantation of combined optical fiber and tetrode recording assembly. Rats were anesthetized with isoflurane (1–3% in oxygen at 1.0 L/min) and received preoperative (0.03 mg/kg buprenorphine) and postoperative (0.05 mg/kg buprenorphine, 1.0 mg/kg meloxicam) analgesia. Additional care and nutrition were given as needed. A single craniectomy was created above the BLA and intermediate third of the hippocampus (coordinate range: 2.6–5.9 mm posterior and 2.8–5.5 mm lateral to Bregma; Paxinos and Watson, 1998) for a unilateral infusion and implantation in the right hemisphere. The viral vector containing channelrhodopsin and reporter fluorophore [AAV_5_-CaMKII-hChR2(H134R)-EYFP; University of North Carolina Vector Core] was infused using a stereotaxic frame (Kopf Instruments) and syringe pump (Hamilton Company). The virus was infused through a 33-gauge needle into the BLA (coordinates: 3.5 mm posterior, 5.1 mm lateral, 8.9 mm ventral to Bregma) at 150 nL/min for a total volume of 500 nL. The needle was left in place for 10 min before withdrawal to allow the virus to diffuse into the surrounding tissue.

After withdrawal of the needle, the recording assembly containing a fixed optical fiber with a ceramic ferrule (200/230 nm, 0.66 NA; Plexon, Inc.) and independently moveable nichrome tetrodes was implanted. Tetrodes were spun with 12.5 nichrome wire (California Fine Wire or Sandvik) and plated with gold to reduce the impedance to approximately 200 kΩ at 1 kHz. The optical fiber was fixed in the recording assembly so that it was positioned directly above the BLA (coordinates: 3.5 mm posterior, 5.1 mm lateral, 8.4 mm ventral to Bregma) with the base of the recording assembly at the surface of the exposed brain. Tetrodes targeting the BLA were glued to the optical fiber to target 0.25–0.75 mm below the fiber tip. Tetrodes targeting the hippocampus were each controlled by a separate driver. They were targeted at the intermediate third of the hippocampus (coordinates range: 4.5–5.9 mm posterior, 2.9–5.4 lateral mm to Bregma), since the intermediate CA1 receives direct projections from the BLA (Pikkarainen et al., 1999; Pitkänen et al., 2000; Petrovich et al., 2001) and has been shown to be involved in memory enhancement by brief electrical stimulation to the BLA (Bass et al., 2014; Bass and Manns, 2015). The rat was grounded by a wire attached to a stainless-steel screw, which was implanted in the skull midline over the cerebellum. This ground screw also served as the reference for LFP recordings. After a minimum of 1-week recovery, tetrodes were slowly lowered into the pyramidal cell layer of the CA1 over the following weeks (recording tetrodes in BLA were fixed to the optical fiber). No tetrodes were moved within 24 h of stimulation and recording.

### Optogenetic Stimulation

Testing occurred no sooner than 4 weeks post-surgery to allow sufficient time for viral transfection and opsin expression. All stimulation occurred on awake rats as they freely explored a 30-cm diameter circular recording platform bordered by an approximately 7-cm wall. Stimulation events were triggered by the experimenter no less than 10 s apart. Stimulation was never dependent on a particular behavioral state other than ensuring that the rat was awake throughout the experiment; the experimenter was not directly observing the animal during stimulation. Light was produced by a compact LED at 465 nm (blue) or 740 nm (near-infrared) (Plexon, Inc.). The blue LED produced light within the excitation spectrum of channelrhodopsin, and the near-infrared LED produced light outside of the excitation spectrum, a method documented to act as a reliable control (Blumberg et al., 2016; Klavir et al., 2017). The LED was connected to the optical fiber’s ferrule on the recording assembly by an armored patch cable (200/230 nm, 0.5 NA) and ceramic coupler (Plexon, Inc.).

Stimulation included several parameter conditions, the order of which was randomized across rats. All rats experienced a least 20 bouts of each condition. Rats received stimulation in the following conditions: (1) 1-s blue light at 8 Hz, (2) 1-s blue light at 20 Hz, (3) 1-s blue light at 50 Hz, (4) 1-s blue light in bursts of four 50 Hz pulses every 1/8th second (50/8 Hz), (5) 5-s blue light at 50 Hz, (6) 5-s blue light at 50/8 Hz, and (7) 1-s near-infrared light at 20 Hz. Stimulation parameters were chosen to mimic theta (8 Hz), slow gamma (50 Hz), theta–gamma comodulation (50/8 Hz), and a frequency (20 Hz) known to reliably evoke responses from ChR2(H134R). All light pulses were of 5 ms duration. Power at the optical fiber tip was approximately 11 mW for the blue LED and 7 mW for the near-infrared LED.

### Histology

Prior to euthanasia, the location of each tetrode was marked by passing 20–40 μA current for 10–30 s through a single wire of the tetrode. Rats were injected with an overdose (0.5 mL) of Euthanasia-III Solution (Med-Pharmex) after being anesthetized with isoflurane. They were then transcardially perfused with isotonic saline followed by neutral buffered formalin 10% (Harleco). Brains were extracted, post-fixed in neutral buffered formalin 10% for 24 h, and submerged in a 30% sucrose solution until saturated. Brains were sectioned on a freezing stage microtome at 40 μM thickness and stored in 0.1 M phosphate buffer. All sections were mounted on slides coated with gelatin and chromium potassium sulfate dodecahydrate (Fisher Scientific). For verification and localization of virus expression, slides were covered with Vectashield with DAPI (Vector Laboratories), and cover slipped. Expression of channelrhodopsin was inferred by the expression of the conjugated fluorophore, observed on an epifluorescence microscope for regional expression and on a confocal microscope for cell body and fiber identification. BLA tetrodes were localized by staining for acetylcholinesterase, which robustly stains the basal nucleus of the BLA. Hippocampal tetrodes were localized under light microscopy following a Nissl stain (cresyl violet).

### Data Acquisition and Analysis

Local field potentials (LFPs) were recorded from tetrodes in the BLA and hippocampus with a sampling rate of 1.5 kHz and were filtered from 1 to 400 Hz. The LFP from one tetrode in the pyramidal layer of CA1 and one tetrode in the BLA was used for each rat. Spiking data were not analyzed due to too few well-isolated single units. All data were obtained with the NSpike data acquisition system^1^. Analyses were performed in MATLAB (MathWorks) using custom scripts and the Chronux toolbox (Bokil et al., 2010). Power of the BLA and CA1 LFPs was estimated using a multitaper fast Fourier transform similar to previous reports (Bass and Manns, 2015; Trimper et al., 2017). The modulation index (MI) for phase-amplitude cross-frequency coupling (i.e., comodulation) was calculated as previously described (Tort et al., 2009).

For all analyses, results were averaged within a rat across all trials of a given condition, and then the data from all rats were averaged. For some analyses, a rat’s data from a single stimulation condition were normalized prior to averaging across rats to demonstrate more clearly the impact of stimulation. In particular, for analyses of average stimulation-evoked LFPs in the time domain, LFPs from 4 s before stimulation onset to 5 s after stimulation offset were Z-transformed based on the mean and standard deviation of each single trial sweep. For spectral analyses in the frequency domain, FFT analyses were conducted on the raw LFPs. Absolute power is shown in spectrograms. However, for plots of moving-window spectrograms, estimates of power were normalized (Z-transformed) to a pre-stimulation baseline period (from -2 to -1 s before the onset of stimulation) to visualize more clearly the impact of stimulation.

Statistical significance was determined using a random permutation approach in which LFP data from the stimulation and baseline periods were randomly shuffled 1,000 times. All analyses were recalculated for each random shuffle, and statistical significance was defined as metrics falling outside the 95th percentile of the distribution obtained from the random shuffles. More specifically, power plotted in spectrograms was analyzed with a cluster-based permutation in which clusters were defined as frequency ranges in which the power values were greater than 2.5 standard deviations above or below the mean of the data. Only clusters spanning more than 1 Hz were considered. The random cluster permutation distribution included only the largest cluster from each random permutation. Clusters (frequency ranges) in the original data that were outside the 95th percentile of the random cluster distribution were labeled as statistically significant. This cluster-based approach was used because it preserves the overall alpha level (Maris and Oostenveld, 2007). For 5-s stimulation conditions, the same cluster-based random permutation approach was used for each second of stimulation. Power during seconds 1–5 were compared against baseline activity (-2 to -1 s before stimulation) independently to determine how the response to stimulation developed over time. Changes in comodulation were analyzed in a pre-determined theta–gamma range (6–10 Hz phase frequency, 30–55 Hz amplitude frequency). A random permutation analysis of variance was used to determine the significance of the effect of stimulation condition on the pre-defined theta–gamma comodulation. Specifically, the variance was defined as summed variance to the mean across stimulation conditions. The mean was the average comodulation index across conditions, and the variance was the difference between the comodulation during an individual condition and the mean. The random permutation was constructed by shuffling the condition labels for each stimulation bout. The resulting variance from the mean of the shuffled data populated the random permutation distribution. The effect of stimulation condition on comodulation was considered significant if the variance of the original data fell outside the 95th percentile of that distribution.

## Results

### Histological Verification of Stimulation and Recording Locations

**FIGURE 1:**
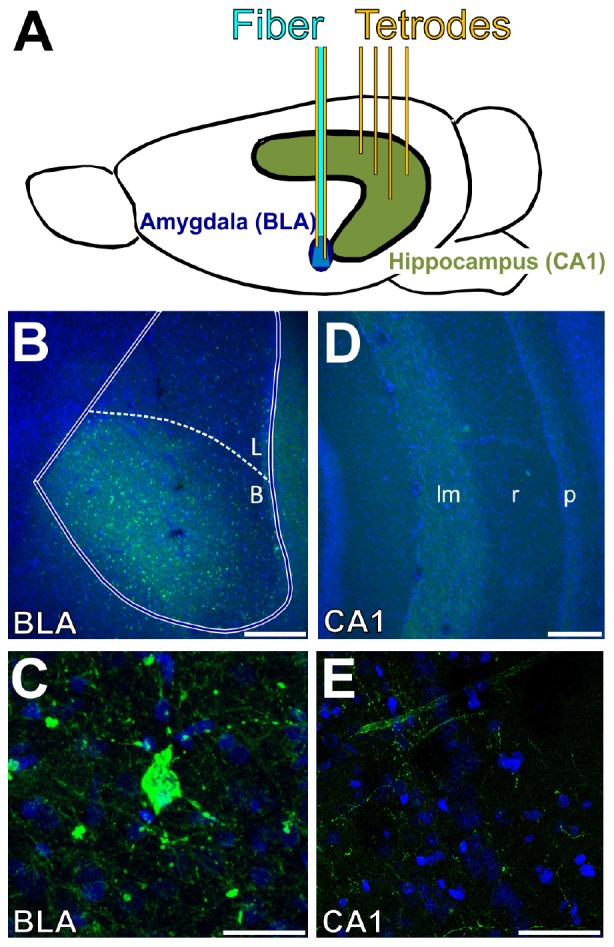
Location of opsin expression, optogenetic stimulation, and tetrode recording. **(A)** Tetrodes (yellow) were lowered into the CA1 subregion of the intermediate hippocampus (green) and basolateral amygdala (blue). An optical fiber (cyan) was lowered to 0.25–0.75 mm above the target depth in the BLA. **(B–E)** Images of coronal sections of the BLA **(B,C)** and intermediate hippocampus **(D,E)**. Nuclei are shown in blue (DAPI), and eYFP expression (conjugated to the opsin) is shown in green. **(B)** CaMKII^+^ neurons were transfected in the basal nucleus of the amygdala. **(C)** The opsin was preferentially expressed in the cell bodies of the BLA. **(D)** Projections from the BLA also expressed the viral vector in the lacunosum-moleculare layer of CA1 in the intermediate hippocampus. **(E)** Hippocampal expression of the opsin was restricted to axonal projections; no cells were labeled in the hippocampus. Scale bars are 250 μm in **(B,D)**, and 30 μm in **(C,E)**. BLA, basolateral complex of the amygdala; B, basal nucleus; L, lateral nucleus; lm, lacunosum-moleculare; r, radiatum; p: pyramidale.

Postmortem histological analysis verified placement of the optical fiber dorsal to the BLA, expression of channelrhodopsin in the BLA, and location of recording tetrodes in the BLA and CA1 of the intermediate hippocampus. Figure 1 shows a schematic of the stimulation and recording approach as well as example histology. The tip of all unilaterally implanted optical fibers was confirmed to be positioned 0.2–0.6 mm dorsal to the BLA. In all six rats, the viral vector transfected cell bodies in the BLA, as evidenced by punctate expression of the fluorophore conjugated to the opsin (Figure 1D). Expression in the amygdala was restricted to the BLA. In two rats, the viral vector spread to a modest degree into the adjacent piriform cortex. However, the off-target neurons were largely outside the cone of light (with a 0.66 numerical aperture, light was emitted from the optical fiber at 29.0°), since the majority of labeled neurons in the piriform cortex were in the dorsal endopiriform nucleus (Paxinos and Watson, 1998). Thus, the impact of light stimulation was largely restricted to neurons in the BLA in all rats. In all rats, fluorophore-labeled fibers were visible in the temporal half of the hippocampus, particularly in the lacunosum-moleculare layer of CA1 and subiculum (Figure 1E), consistent with past studies showing projections from the BLA to hippocampus terminating in this specific area (Pitkänen et al., 2000; Wang and Barbas, 2018). Analyses of neural data focused on LFPs recorded from single electrodes in the BLA and CA1 in order to align the current results with past results from humans and rats (Bass et al., 2012, 2014; Bass and Manns, 2015; Inman et al., 2018) and because too few well-isolated single neurons were recorded to permit spiking analyses. All six rats had at least one tetrode positioned in the basolateral nucleus, and five rats had at least one tetrode positioned in the pyramidal layer of the CA1. The pyramidal layer was selected as a target layer to allow for comparison with past studies (Trimper et al., 2014; Bass and Manns, 2015), and because it could be localized at the time of recording by the presence of putative pyramidal neuron spiking. Analyses of LFP data from the BLA thus included six rats, whereas analyses of data from CA1 included five rats.

### Effects of 1-s Optogenetic BLA Stimulation on BLA and CA1 LFPs

**FIGURE 2:**
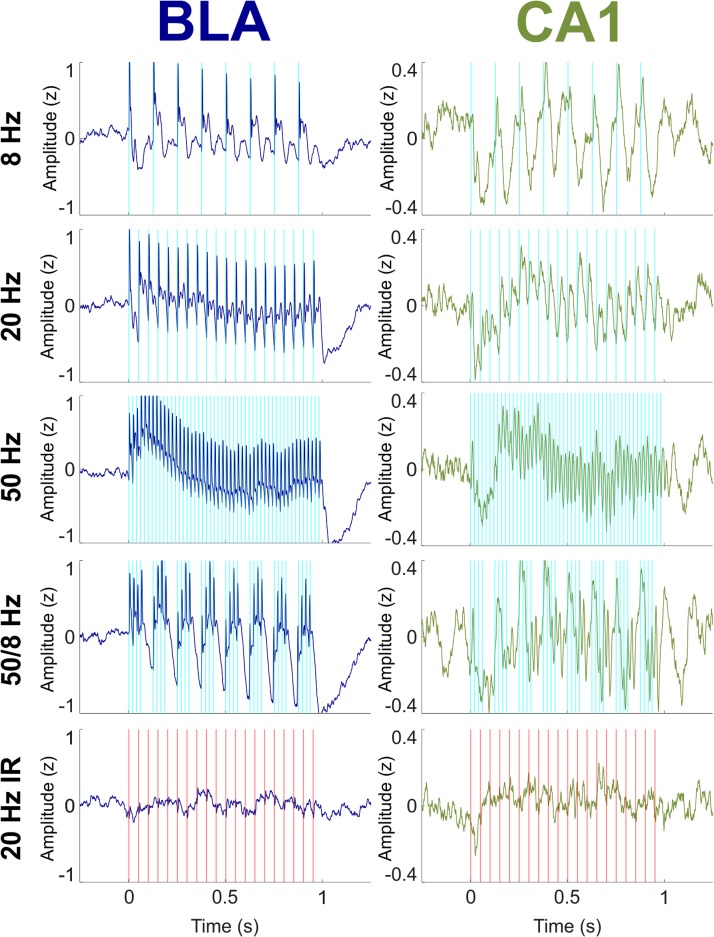
Effects on BLA and CA1 LFPs of optogenetic BLA stimulation with blue (experimental) light at 8, 20, 50, and 50/8 Hz and with near infrared (IR; control) light at 20 Hz. LFPs were Z-transformed within conditions for each rat and then averaged across all rats. **(Left)** Evoked responses in the BLA (blue) tracked stimulation at all frequencies. **(Right)** LFPs in the CA1 (green) tracked stimulation at all frequencies with more regular oscillatory activity than what was seen in the BLA.

Figure 2 shows the mean normalized (Z-transformed) LFP in the BLA and CA1 during 1-s bouts of optical stimulation of the BLA. Stimulation was delivered up to 70 times per condition (mean number of stimulations per condition = 39.9; range = 20–70) over the course of multiple recording sessions for each rat (mean number of recording sessions per rat = 2.83; range = 1–4). The optical stimulation was a blue 465-nm light delivered at 8, 20, or 50 Hz, or as bursts of four 50-Hz pulses delivered every 1/8th second (“50/8 Hz”). A control condition consisted of 1 s of 20-Hz near-infrared 740-nm light to the BLA, a wavelength known to be outside the excitation spectrum of channelrhodopsin (Mattis et al., 2012). Optical stimulation with blue light in the 8, 20, 50, and 50/8 Hz conditions evoked large responses of the same frequencies in the LFPs in the BLA (*Z*-scores ranged from about -1 to +1 across conditions) and moderate responses of the same frequencies in the LFPs in CA1 (*Z*-scores ranged from about -0.4 to +0.4 across conditions). Evoked responses in both regions appeared to cease soon after termination of stimulation in each condition. The control 20-Hz near-infrared optical stimulation did not evoke appreciable responses in either the BLA or CA1. These results suggest that optical stimulation of the BLA with blue light was capable of evoking frequency-matched responses in both the BLA and CA1 and that the evoked responses were a direct result of activation of the opsin, not an optoelectronic artifact or an artifact of the recording system.

**FIGURE 3:**
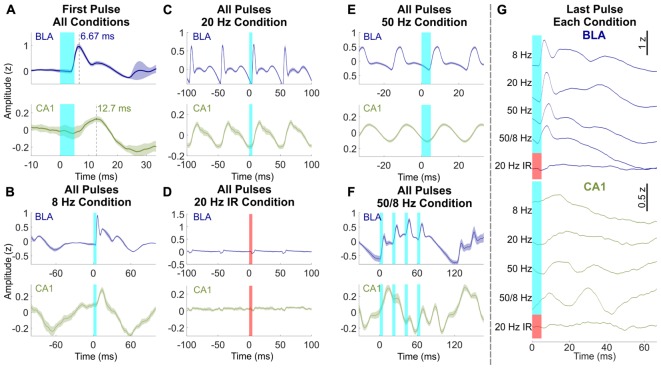
Effects of BLA stimulation pulses differed between BLA and CA1. **(A)** Response latencies to the first light pulse of all conditions in the BLA (blue, top) and CA1 (green, bottom). Time to LFP peak response was 6.67 ms in the BLA and 12.7 ms in CA1. **(B–F)** Averaged LFP across all light pulses within the 8 Hz, 20 Hz, 20 Hz near-infrared, 50 Hz, and 50/8 Hz stimulation conditions, respectively (averaged across bursts of 50 Hz pulses for **F**). Responses in the BLA were characterized by fast activity that was similar across conditions, whereas oscillatory activity in CA1 was dependent on stimulation frequency. Maximal gamma activity preferentially occurred at the peak of theta in the BLA and trough of theta in CA1. **(G)** Response in the BLA and CA1 after the last pulse of each condition. 50/8 Hz stimulation produced persistent gamma in CA1 after termination of stimulation.

Although the overall responses to 1 s of light stimulation were similar in the BLA and CA1, a closer inspection highlighted important differences between the regions. Figure 3 shows mean normalized evoked responses in the BLA and CA1 to individual pulses of light delivered to the BLA. Averaged across all blue light stimulation conditions, the latency from onset of the first light pulse in each bout of stimulation to the initial peak of evoked response was 6.67 ms in the BLA, which reflects the response time of the opsin to light stimulation (Mattis et al., 2012). The latency to the initial peak was 12.7 ms in CA1 (Figure 3A), a 6.03 ms difference, suggesting that the responses recorded in CA1 were neither triggered directly by the light nor conducted passively by brain volume but instead were evoked by monosynaptic connections from the BLA. Averaging across all light pulses separately for each condition shows additional differences between the responses in the BLA and CA1 (e.g., averaging across all 8 pulses in the 8 Hz condition). BLA LFPs were characterized by evoked responses of the same width (approximately 9 ms) in the 8, 20, 50, and 50/8 Hz conditions (Figure 3B–F). The initial evoked responses were followed by smaller responses in the fast gamma range (60–120 Hz), which is a prominent frequency band in the amygdala (Amir et al., 2018; Feng et al., 2019). In contrast, CA1 LFPs during stimulation with blue light displayed a more continuous waveform that had a sawtooth shape for 8 and 20 Hz conditions and a sinusoidal shape for 50 Hz stimulation (Figure 3B–D). For the 50/8 Hz condition, LFPs in the BLA and CA1 both showed 8 Hz and 50 Hz components in the shape of the response to the four 50-Hz pulses delivered every 1/8th second (Figure 3F). However, the 8 Hz response was out of phase between the BLA and CA1, and the 50-Hz response was delayed by at least a full 50 Hz cycle in CA1 compared to the BLA. Thus, the 50 Hz responses were largest on the rising slope of the 8 Hz wave in the BLA but largest on the falling slope of the 8 Hz wave in CA1. The differences in LFP responses between the BLA and CA1 suggested that stimulation of the BLA modulated activity within the hippocampus above and beyond a simple recapitulation of the stimulation effects in BLA. LFPs in both regions showed a small artifact during 20 Hz near-infrared stimulation, but the artifacts were the opposite polarity and occurred at a shorter delay as compared to those produced by blue light stimulation (Figure 3D).

A final comparison of waveforms between the BLA and CA1 during BLA stimulation focused on the average normalized response following the last light pulse in each bout of stimulation (Figure 3G). LFPs in the BLA following the last pulse of light were similar to the previous analyses of LFPs averaged across all light pulses in a condition (Figure 3B–F), and LFPs averaged across the first light pulse of each condition (Figure 3A). For example, for each condition, the delay of initial peak responses of the BLA LFP following the final BLA pulse was similar (range = 6.67–8.67 ms) to the average delay in response to the first pulse (6.67 ms) and was followed by fast, small amplitude activity in each case. In contrast, LFPs responses in CA1 following the last pulse of light differed across stimulation conditions. The times to initial peak response in CA1 following the final BLA pulse were 14.0, 19.3, 16.0, and 9.33 ms for 8, 20, 50, and 50/8 Hz conditions, respectively. In addition, a full extra cycle of slow gamma activity persisted in CA1 following the last pulse of 50/8 Hz stimulation and, to a lesser extent (and with different timing) following the last pulse of 50 Hz stimulation. The frequency-dependent persistent activity in CA1 supports the characterization of responses in the CA1 as oscillations rather than concatenated evoked responses, particularly for the 50/8 Hz stimulation condition.

### Effects of 1-s Optogenetic BLA Stimulation on Power Spectra in the BLA and CA1

**FIGURE 4:**
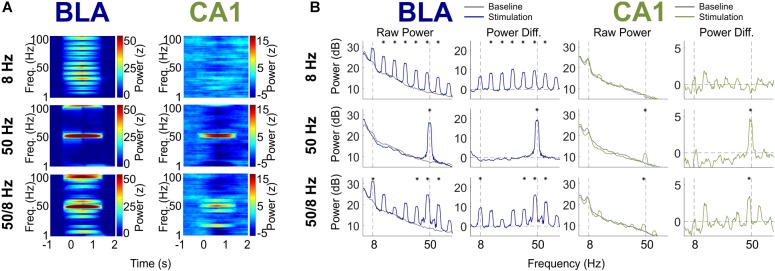
BLA and CA1 LFP Power in response to optogenetic BLA stimulation. **(A)** Moving window spectrogram of power around the stimulation event. Power was normalized to the baseline period for clarity. **(B)** Spectrogram of power during stimulation, displayed as absolute decibels (left) and with the baseline period subtracted (right). Asterisks indicate frequency clusters that differed significantly between stimulation and baseline periods.

Figure 4 shows the power spectra for the 8, 50, and 50/8 Hz conditions following a multitaper fast Fourier transform (FFT) of the BLA and CA1 LFP traces (see section “Materials and Methods” for analysis details, including testing for statistical significance). The results are shown as normalized (Z-transformed) moving window power spectrograms as well as standard power spectrograms to illustrate and statistically evaluate changes in the theta and gamma frequency ranges during stimulation relative to a pre-stimulation baseline. The moving window power spectrograms were calculated using a 1-s sliding window, so power values for a given timepoint contain information from the preceding and following 0.5 s. For LFPs from the BLA, BLA stimulation in the 8 and 50 Hz conditions resulted in increased power in the 8 and 50 Hz frequency ranges (plus harmonics), respectively. The increase in 50 Hz power was statistically significant for the 50 Hz condition, and the increase in power in the 8 Hz harmonic ranges (peaks at 16, 24, 32, 40, 48, and 56 Hz) was statistically significant for the 8 Hz condition. Stimulation in the 50/8 Hz condition resulted in statistically significantly increased BLA power at 8, 40, 48, and 56 Hz. In contrast to the results from BLA LFPs, CA1 LFPs showed power with prominent peaks in the 8 Hz range during the baseline in all conditions but did not show increased power in the 8 Hz range for any stimulation condition. The lack of increase in 8 Hz CA1 power in the 8 Hz stimulation condition contrasts with the clear entrainment of CA1 LFPs at 8 Hz during 8 Hz stimulation (see Figure 2 top right panel, and Figure 3B). Thus, the phase but not the amplitude of ongoing hippocampal theta oscillations appeared to be modulated by 8 Hz BLA stimulation. Stimulation in both the 50 Hz and 50/8 Hz conditions resulted in significantly increased CA1 power in the slow gamma range. However, the peak frequency of CA1 power increase was 50 Hz during 50 Hz stimulation yet 48.7 Hz during 50/8 Hz stimulation, which is closer to a harmonic (48 Hz) of the underlying 8 Hz pattern than 50 Hz. Thus, the 8 Hz entrainment of CA1 LFPs during 8 Hz stimulation and the slightly shifted peak slow gamma power increase in the 50/8 Hz (48.7 Hz rather than 50 Hz) both suggest that the 8 Hz component of BLA stimulation in the 8 Hz and 50/8 Hz conditions did influence CA1 LFPs. Nevertheless, the only statistically significant power increases in CA1 during BLA stimulation in 8, 50, and 50/8 Hz conditions were in the slow gamma range (∼50 Hz).

### Theta–Gamma Comodulation During Optogenetic Stimulation

**FIGURE 5:**
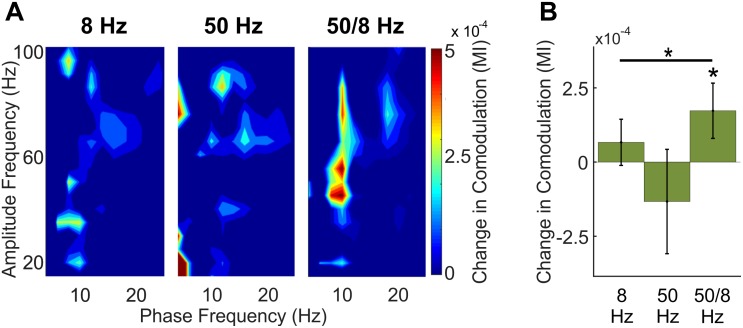
Comodulation within CA1 across BLA stimulation conditions. **(A)** Comodulogram during 1-s BLA stimulation with the baseline period subtracted for clarity. **(B)** Total theta-modulated-slow gamma during stimulation relative to baseline for each stimulation conditions. Asterisks indicate that theta–gamma comodulation was significantly increased during 50/8 Hz stimulation and that theta–gamma comodulation was significantly different across conditions.

A key question was whether optogenetic stimulation of putative BLA glutamatergic projection neurons could increase gamma oscillations in the hippocampus for which the amplitude was modulated by the phase of theta oscillations—the type of phase-amplitude cross-frequency coupling (here referred to in brief as comodulation) known to be important for memory (Tort et al., 2009; Shirvalkar et al., 2010; Trimper et al., 2014). Figure 5 shows comodulation in CA1 during BLA stimulation relative to baseline in the 8, 50, and 50/8 Hz conditions. Only stimulation in the 50/8 Hz condition statistically significantly (*p* < 0.05 per a random permutation analysis; see section “Materials and Methods”) increased theta–gamma comodulation relative to baseline [mean modulation index (MI) = 0.67 × 10^-4^, -1.33 × 10^-4^, and 1.73 × 10^-4^, for 8, 50, and 50/8 Hz conditions, respectively]. In addition, the stimulation condition was a statistically significant factor (*p* < 0.05 per a random permutation analysis) in theta–gamma comodulation across 8, 50, and 50/8 Hz conditions (see section “Materials and Methods” for analysis details). Thus, theta-modulated gamma oscillations were increased in CA1 only when BLA stimulation included both theta and gamma frequencies.

### Temporal Effects of Optogenetic Stimulation

**FIGURE 6:**
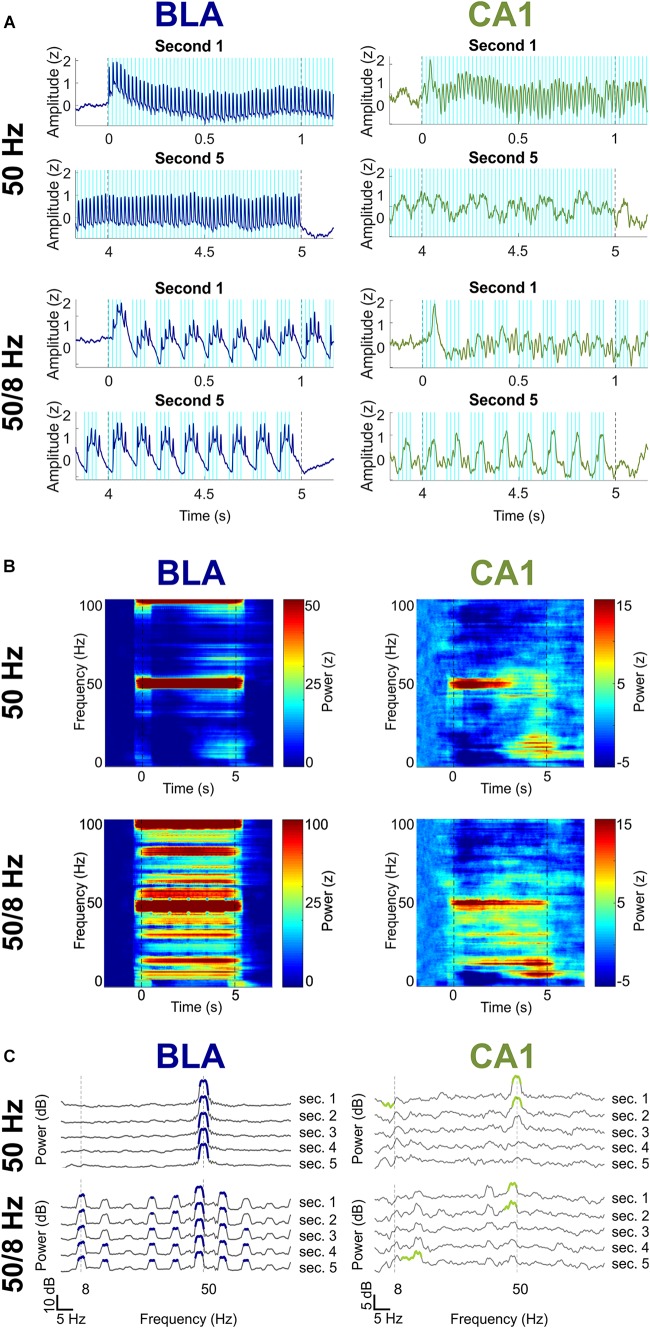
Effects of 5-s 50 and 50/8 Hz stimulation on the BLA (blue) and CA1 (green). **(A)** Averaged BLA and CA1 LFPs in the first and last seconds of 5-s BLA stimulation. LFPs were Z-transformed and averaged across rats. **(B)** Moving window power spectrogram during 50 and 50/8 Hz 5-s stimulation, normalized to the baseline period for clarity. **(C)** Power spectrogram of second 1 (top) to second 5 (bottom), normalized against the baseline period. Frequency ranges that differed significantly between stimulation and baseline are highlighted in blue (BLA) and green (CA1).

The final question was whether longer bouts of BLA stimulation might elicit larger or different responses as compared to 1 s of simulation. Figure 6 shows activity in the BLA and CA1 during 5 s of BLA stimulation in the 50 Hz and 50/8 Hz conditions (*n* = 3 for these data). The BLA LFPs did not appreciably change over the 5 s of BLA stimulation in either condition. In contrast, CA1 LFPs substantially changed from the first to the fifth second of stimulation, such that prominent fast oscillatory activity at the beginning of stimulation was almost completely replaced by slow oscillatory activity by the end of stimulation. For the 50 Hz stimulation condition, the 50 Hz CA1 oscillations in the first second returned to baseline levels and were largely replaced by slow oscillations in the 8 and 16 Hz ranges by the fifth second of stimulation. For the 50/8 Hz condition, CA1 oscillations in the 48-Hz range decreased moderately and CA1 oscillations in 8 and 16 Hz ranges increased markedly from the first to fifth second of BLA stimulation. In the BLA, 5-s BLA stimulation at 50 Hz stimulation evoked statistically significant increases in gamma power for each of the 5 s. In addition, 5-s BLA stimulation at 50/8 Hz stimulation evoked statistically significant increases in both theta (plus harmonics) and gamma power for each of the 5 s of stimulation. Thus, longer bouts of BLA stimulation resulted in temporally static responses in BLA LFPs but temporally dynamic responses in CA1 LFPs. It is unclear why the 5-s BLA stimulation resulted in different hippocampal activity as compared to 1 s of BLA stimulation. In any case, the present results suggest that brief (less than 2 s) optogenetic 50/8 Hz stimulation of the BLA would be most likely to elicit hippocampal oscillatory states thought to be beneficial to memory.

## Discussion

Brief optogenetic stimulation of the BLA at 8, 20, 50, and 50/8 Hz reliably elicited responses at matching frequencies in LFPs recorded in the BLA and CA1 in freely exploring rats. However, stimulation responses in CA1 differed from responses in the BLA in several ways. As compared to the responses in the BLA, the responses in CA1 across conditions were delayed by approximately 6 ms, displayed more continuous (sinusoidal or sawtooth) waveforms, and showed dynamic oscillatory changes across longer bouts (5 s) of stimulation. Thus, CA1 LFPs showed responses during BLA stimulation that broadly resembled neuronal oscillations, whereas BLA LFPs showed responses that resembled concatenated evoked responses. Moreover, the responses in CA1 LFPs to BLA stimulation differed between the 8, 50, and 50/8 Hz stimulation conditions, which were the focus of the current study. In particular, BLA stimulation in the 50 and 50/8 Hz conditions led to increased power close to 50 Hz in CA1, but none of the 8, 50, and 50/8 Hz conditions led to increased CA1 power in the 8 Hz range, despite 8 Hz BLA stimulation clearly entraining the phase of the ongoing 8 Hz theta oscillation in the hippocampus. A key finding was that 1 s of 50/8 Hz BLA stimulation preferentially increased in CA1 LFPs 50 Hz oscillations for which the amplitude was modulated by the phase of the 8 Hz oscillations, a type of phase-amplitude cross-frequency coupling (theta–gamma comodulation) known to be important for good memory. Thus, artificial stimulation of the BLA appears to be capable of increasing in the hippocampus neuronal oscillations that resemble endogenous oscillatory states that are thought to benefit memory formation. The results are discussed in more detail below.

### BLA Projections to CA1 Were Among Many Potential BLA Projections Activated by Stimulation

The BLA includes the basal, lateral, and accessory basal nuclei (Sah et al., 2003). Neurons in these nuclei send axons to regions essential for declarative memory, including the hippocampus, entorhinal cortex, and perirhinal cortex, as well as to many other regions of the brain and to other amygdalar nuclei (Pitkänen et al., 1995, 2000; Savander et al., 1996; Sah et al., 2003). Thus, optogenetic stimulation of putative glutamatergic BLA projection neurons could have influenced neuronal activity in the hippocampus both directly and indirectly. One potential pathway mediating the indirect effects of BLA stimulation on the hippocampus is the pathway from perirhinal cortex to entorhinal cortex to hippocampus (Burwell and Amaral, 1998; Witter and Amaral, 2004). For example, activation of the amygdala is thought to facilitate information transfer from the perirhinal cortex to the entorhinal cortex, which in turn would influence the input to the hippocampus (Kajiwara et al., 2003; Paz et al., 2006). In addition, stimulation of the BLA-entorhinal cortex pathway was previously found to enhance hippocampal-dependent memories (Wahlstrom et al., 2018). Additional support for the importance of this perirhinal-entorhinal pathway comes from past studies showing that BLA stimulation modulated hippocampal LTP in the dentate gyrus (Abe, 2001; Akirav and Richter-Levin, 2002; Vouimba and Richter-Levin, 2005), which receives input from the entorhinal cortex but not from the BLA (Pitkänen et al., 2000; Witter and Amaral, 2004). As such, the BLA likely normally engages indirect pathways to influence hippocampal activity and to modulate memory.

Nevertheless, the current results indicated that direct BLA-CA1 projections were an important pathway through which optogenetic stimulation of BLA neurons influenced hippocampal activity. Infusions of the viral vector specifically targeted neurons in the posterior portion of the basal nucleus in the BLA, a region previously found to have strong direct projections to CA1 (Pitkänen et al., 2000). Postmortem histology in the present study confirmed expression of the opsin and reporter fluorophore in cell bodies in this nucleus as well as in fibers in the lacunosum-moleculare layer of intermediate CA1, consistent with the laminar profile of past anatomical studies of direct basal nucleus projections to CA1 (Pitkänen et al., 2000; Wang and Barbas, 2018). Thus, CA1 recording tetrodes were positioned near the soma (in pyramidale) of pyramidal neurons likely receiving synaptic inputs on their apical dendrites (in lacunosum-moleculare) from opsin-containing BLA neurons. Further, the short delay (6.03 ms) observed between BLA and CA1 responses to initial pulses of BLA stimulation strongly supported the involvement of this monosynaptic pathway. Previous studies have also shown that manipulation of this direct pathway was sufficient to drive behavioral changes (Rei et al., 2015; Huff et al., 2016). Taken together, the results suggested that the direct projection from BLA to CA1—although only one of many BLA projections—was important for the hippocampal responses increased by optogenetic BLA stimulation.

### BLA Stimulation Modulated Neuronal Oscillations in CA1

The pattern of CA1 LFP activity in response to BLA stimulation reflected more than a concatenation of depolarizing events. Instead, CA1 LFPs responded to 1-s BLA stimulation in a manner more characteristic of neuronal oscillations, evidence for synaptic transmission that included (but was not limited to) direct BLA to CA1 projections. Specifically, LFP activity in CA1 during each of the 1-s BLA stimulation conditions showed rhythmic sinusoidal or sawtooth waveforms that corresponded to the stimulation frequency. In contrast, LFP activity in the BLA showed a sharp evoked response to each light pulse that was disconnected from preceding responses and was unrelated to stimulation frequency. One possible source of the differences between responses in BLA and CA1 LFPs was that the CA1 LFP responses may have been shaped by the low-pass frequency filtering that occurs during synaptic transmission, particularly in the case of synapses on the distal portion of apical dendrites of pyramidal neurons (Buzsáki et al., 2012), as was likely in the present study. Indeed, it is possible that synaptic transmission between regions is generally important in translating the effects of artificial stimulation to effects more reminiscent of endogenous activity. Nevertheless, the emergence of rhythmic oscillatory activity in CA1 LFPs likely also reflected circuit dynamics in the hippocampus. Possible examples include local excitatory-inhibitory interactions between CA1 pyramidal neurons and interneurons (Buzsáki and Wang, 2012) and rhythmic inputs to the hippocampus from a number of brain regions (Buzsáki, 2002). Thus, direct BLA to CA1 projections were likely key to initiating CA1 LFP responses to stimulation, but the emergence of oscillatory activity in CA1 also likely depended on other intra-hippocampal and extra-hippocampal influences on CA1 activity.

One of these main influences appeared to be ongoing theta oscillations in the hippocampus. Theta (∼8 Hz) oscillations in the hippocampus are prominent and are thought to emerge from a number of influences, including pacemaker inputs from medial septum and entorhinal cortex, from periodic activity of local interneurons, and from resonance properties of pyramidal neurons (Buzsáki, 2002). In the present study, theta power in CA1 LFPs was high at baseline, and neither 1-s BLA stimulation at 8 Hz nor at 50/8 Hz increased theta power in CA1 despite producing a large increase in theta power in BLA LFPs. However, the phase of CA1 theta oscillations appeared to reset and become strongly entrained to the 8 Hz component of both 8 and 50/8 Hz BLA stimulation. That is, hippocampal oscillations in the theta band were still modulated by BLA stimulation, even without significant increases in CA1 theta power.

### Theta-Modulated 50-Hz BLA Stimulation Was Necessary to Increase Theta-Modulated Gamma Oscillations in CA1

A main question motivating the present study was whether BLA stimulation combining theta and gamma frequencies was needed to increase in the hippocampus gamma oscillations for which the amplitude was modulated by the phase of theta, the type of theta–gamma comodulation that is normally observed in the hippocampus (Bragin et al., 1995; Buzsaki et al., 2003). An alternate possibility was that continuous 50 Hz BLA stimulation would interact with endogenous hippocampal theta oscillations to also produce theta-modulated gamma oscillations. Another possibility was that 8 Hz BLA stimulation would modulate extant hippocampal gamma oscillations. Finally, it had been possible that 50/8 Hz BLA stimulation would misalign with existing theta and gamma oscillations in the hippocampus and not result in theta-modulated gamma oscillations in CA1. In short, it was possible that all or none of the stimulation conditions of interest would increase theta–gamma comodulation in the hippocampus. Nevertheless, the results of the current study showed that theta–gamma comodulation within CA1 was significantly increased during 50/8 Hz stimulation but not 50 Hz stimulation or 8 Hz stimulation. The results are important because hippocampal gamma oscillations are normally modulated by theta phase and because hippocampal theta–gamma comodulation is a neural state previously observed to correlate with successful encoding and retrieval of hippocampal memory (Tort et al., 2009; Shirvalkar et al., 2010; Trimper et al., 2014). Indeed, one hypothesis about amygdala-mediated declarative memory enhancement is that activation of the BLA elicits theta-modulated gamma oscillations in the hippocampus, which in turn promotes spike-timing dependent plasticity for recently active synapses (Manns and Bass, 2016).

### Comparing the Effects of Optogenetic BLA Stimulation to Those of Electrical BLA Stimulation

Several previous studies in rats (Bass et al., 2012, 2014; Bass and Manns, 2015) and humans (Inman et al., 2018) observed improved 24-h recognition memory performance for neutral objects when the initial presentation of the objects was immediately followed by 1 s of 50/8 Hz electrical stimulation of the BLA. One of the studies in rats (Bass and Manns, 2015) also recorded neuronal activity in the intermediate hippocampus at the time of stimulation and observed increased coherence (both field-field and spike-field) between CA3 and CA1 in the slow gamma range (theta–gamma comodulation was not reported). The similarity in increased hippocampal gamma oscillations between this prior study and the present study suggests that activation of the BLA via either electrical or optogenetic stimulation can elicit oscillatory states in the hippocampus that favor memory. Nevertheless, the mechanisms of BLA activation likely differed between optogenetic and electrical stimulation. For example, optogenetic stimulation in the present study more preferentially depolarized glutamatergic cell bodies in the transfected area (though the depolarization of any neuron may have been stochastic rather than deterministic for the 50-Hz stimulation; Cardin et al., 2009; Weitz et al., 2015), whereas electrical stimulation in prior studies would have stimulated all neuron types as well as fibers of passage (Histed et al., 2009). Perhaps reflecting these differences, electrical pulses delivered to the BLA in the prior study (Bass and Manns, 2015) resulted in initial evoked responses in the hippocampus after a delay (24 ms) that suggested a polysynaptic effect of stimulation rather than the monosynaptic effect thought to be important in the present study. One possibility is that electrical stimulation of the BLA more strongly engaged the BLA-perirhinal/entorhinal-hippocampus pathway, whereas optogenetic stimulation of the BLA more strongly engaged the BLA-hippocampus pathway. If so, the results would suggest that activation of either pathway would be sufficient to produce memory-promoting oscillatory states in the hippocampus characterized by slow gamma oscillations.

## Conclusion

The ability of 50/8 Hz optogenetic BLA stimulation to elicit theta–gamma comodulation in the hippocampus provides important insights into how the amygdala may modulate the hippocampus to prioritize memories with affective salience. Memory modulation should benefit some memories more than others if important moments are to be remembered better than unimportant moments. Thus, amygdala stimulation will likely need to be temporally specific to prioritize memories effectively. Indeed, consideration of temporal specificity will be important for any possible future therapeutic interventions and may be one explanation for the mixed results of past memory studies targeting amygdala activity (Agren, 2014; Taylor and Torregrossa, 2015). In addition, previous experiments have shown that the effects of direct hippocampal stimulation can depend on the neural state immediately prior to stimulation, which is possibly why closed-loop stimulations of the hippocampus have sometimes enhanced memory (Berger et al., 2011; Hampson et al., 2012, 2018; Ezzyat et al., 2017), whereas open-loop stimulations (i.e., delivered irrespective of ongoing activity) typically impair memory (Lacruz et al., 2010; Jacobs et al., 2016). In contrast to these studies of direct hippocampus stimulation, 50/8 Hz electrical stimulation of the BLA has reliably improved memory even when stimulation onset was not dependent on ongoing neuronal activity (Bass et al., 2012, 2014; Bass and Manns, 2015; Inman et al., 2018), perhaps because BLA stimulation was able to reset the phase of ongoing theta oscillations, as observed with optogenetic BLA stimulation in the present study. Finally, the anatomical specificity of BLA stimulation will be an additional important consideration moving forward. Future experiments using optogenetic stimulation of specific BLA projections (e.g., BLA to hippocampus) will be required to determine how projection-specific stimulation of the BLA might differentially impact hippocampal activity or memory performance. Indeed, it is an open question as to whether the glutamatergic neuron-specific optogenetic stimulation used in the present study would result in similar memory enhancement as observed in past studies using electrical stimulation of the BLA.

## Data Availability

The datasets generated for this study are available on request to the corresponding author.

## Ethics Statement

This study was carried out in accordance with the recommendations of Institutional Animal Care and Use Committee at Emory University. The protocol was approved by the Institutional Animal Care and Use Committee at Emory University.

## Author Contributions

NA designed and performed the experiments, analyzed the data, and wrote the manuscript. JM designed the experiments, analyzed the data, and wrote the manuscript.

## Conflict of Interest Statement

The authors declare that the research was conducted in the absence of any commercial or financial relationships that could be construed as a potential conflict of interest.

## Footnotes

1 nspike.sourceforge.net
